# Speech Outcomes after Delayed Hard Palate Closure and Synchronous Secondary Alveolar Bone Grafting in Patients with Cleft Lip, Alveolus and Palate

**DOI:** 10.1055/s-0044-1787002

**Published:** 2024-06-14

**Authors:** Mona Haj, S.N. Hakkesteegt, H.G. Poldermans, H.H.W. de Gier, S.L. Versnel, E.B. Wolvius

**Affiliations:** 1 Department of Maxillofacial Surgery, Erasmus University Medical Center, Sophia Children's Hospital, Rotterdam, The Netherlands; 2 Department of Otorhinolaryngology and Head and Neck Surgery, Erasmus University Medical Center, Sophia Children's Hospital, Rotterdam, The Netherlands; 3 Department of Plastic and Reconstructive Surgery, Erasmus University Medical Center, Sophia Children's Hospital, Rotterdam, The Netherlands

**Keywords:** cleft lip with or without cleft palate, cleft palate, speech, hard palate closure, alveolar bone grafting

## Abstract

**Background** The best timing of closure of the hard palate in individuals with cleft lip, alveolus, and palate (CLAP) to reach the optimal speech outcomes and maxillary growth is still a subject of debate. This study evaluates changes in compensatory articulatory patterns and resonance in patients with unilateral and bilateral CLAP who underwent simultaneous closure of the hard palate and secondary alveolar bone grafting (ABG).

**Methods** A retrospective study of patients with nonsyndromic unilateral and bilateral CLAP who underwent delayed hard palate closure (DHPC) simultaneously with ABG at 9 to 12 years of age from 2013 to 2018. The articulatory patterns, nasality, degree of hypernasality, facial grimacing, and speech intelligibility were assessed pre- and postoperatively.

**Results** Forty-eight patients were included. DHPC and ABG were performed at the mean age of 10.5 years. Postoperatively hypernasal speech was still present in 54% of patients; however, the degree of hypernasality decreased in 67% ( *p* < 0.001). Grimacing decreased in 27% ( *p* = 0.015). Articulation disorders remained present in 85% ( *p* = 0.375). Intelligible speech (grade 1 or 2) was observed in 71 compared with 35% of patients preoperatively ( *p* < 0.001).

**Conclusion** This study showed an improved resonance and intelligibility following DHPC at the mean age of 10.5 years, however compensatory articulation errors persisted. Sequential treatments such as speech therapy play a key role in improvement of speech and may reduce remaining compensatory mechanisms following DHPC.

## Introduction

Depending on the phenotype and extent of oral cleft, patients may suffer from functional and aesthetic impairments such as transient delay in development of speech and disorders in articulation and resonance as well as maxillary hypoplasia. 1 2

Speech of patients affected with cleft palate is often characterized by resonance and articulation errors. Resonance characteristics are hypernasality (air flow into nasal cavity) and nasal emission (nasal air release). It is affected by a combination of structures of the nasal, pharyngeal, and oral cavities and the balance of sound energy in those cavities. 3 Articulation errors occur because of changed articulation placement due to anatomical anomalies. Consequently, children may develop new motor speech patterns such as compensatory articulation patterns to compensate for these speech disorders and facial grimacing, as an attempt to inhibit nasal air leak by constriction of the nasal/facial musculature. 2 4 A general hypothesis is that in case of prolonged persistence of an anterior palatal defect, these compensatory mechanisms are hard to eliminate at older age. 2 5 6 7 8

The ultimate objective of cleft care is finding a balance between the best intelligible speech and reaching the optimal midfacial growth at skeletal maturity and, at the same time, reducing the burden of care for patients with cleft. Although current literature is still not conclusive on the exact impact of the timing of palatal repair on midfacial growth, the focus of the treatment is increasingly shifted on the enhancement of speech at an earliest age as possible. This is based on the objective that quality of speech may substantially influence a person's psychosocial health and social acceptance and integration in society even at a young age. 9

Although there is a general consensus on the timing of lip- and soft palate closure and alveolar bone grafting (ABG), the timing of hard palate closure (HPC) has been debated for decades and varies worldwide between 6 months and 13 years of age. 10 11 12 13 Some studies advocate that by earlier closure of the hard palate, incorporation of articulatory errors would be prevented resulting in a better speech outcome. 5 8 14

Reports on synchronous delayed hard palate closure (DHPC) and secondary ABG and its influence on resonance and articulation are sparse. This study aims to evaluate the documented changes in resonance and articulatory patterns in individuals with an isolated cleft lip, alveolus, and palate (CLAP) who underwent a two-stage palatal closure with simultaneous closure of the hard palate and secondary ABG.

## Methods

This retrospective cohort study includes all children diagnosed with a nonsyndromic CLAP who underwent a synchronous DHPC and ABG between 9 and 12 years of age from January 2013 to June 2018 at authors' institution. Patients were excluded in case of presence of other congenital deformities or patients with an incomplete documentation of speech assessments.

### Local Cleft Treatment Protocol during Study Period

All patients included in this study were treated according to the local treatment protocol for unilateral CLAP as shown in Table 1 . Soft palate closure was conducted using the Widmaier–Perko technique. Within the first 2 years of life, only observation of spoken language and speech was conducted during multidisciplinary outpatient visits. The first assessment of speech and language development took place at 2 years of age. All speech samples were collected in standardized manner with regard to the setting and recording, according to Sell. 15 Speech assessments were conducted by one speech-language pathologist experienced in cleft care. When the development of speech and language skills was not at an appropriate level or when early speech production was inadequate, speech therapy was recommended. Speech therapy would be performed by speech-language pathologists outside of the hospital with varying experience in the treatment of cleft patients. In case of insufficient progression after speech therapy or when velopharyngeal dysfunction was suspected during perceptual speech assessments, nasoendoscopy was conducted at the age of 4 or when the patient would be cooperative enough. If velopharyngeal insufficiency (VPI) was observed, velopharyngeal repair was recommended at an earlier age. Prosthetic speech appliances were not used in our institutions in this cohort.

**Table 1:** Local treatment protocol during the study period

*Timing*	*Performed procedure*
*3–5 months*	Lip repair (according to the Millard technique), with/without primary correction of the nasal ala and/or placement of grommets when indicated
*9–11 months*	Soft palate closure (according to the Perko technique)
* 9–12 years a*	Simultaneous hard palate closure (according to von Langenbeck) and alveolar bone grafting
*18+ years*	If indicated, orthognathic surgery after completion of facial skeletal growth and secondary nose and/or lip corrections

a Timing of alveolar bone grafting was determined by the extent of the maturity of the canine roots (two-thirds) or the degree of eruption of the lateral incisors.

Standardized speech assessments were conducted before and after HPC and alveolar repair. These were performed using the speech assessment protocol developed by Meijer in collaboration with the Dutch Association for Cleft and Craniofacial Anomalies based on the framework that was devised for the Eurocleft Speech Project 16 (Shaw, 2001 33 ).

The timing of HPC was commonly determined by the eruption state of the canine. If earlier alveolar bone repair would enhance the eruption of the lateral incisor, ABG would be planned based on the development of the lateral incisor and thus would be performed at an earlier stage.

### Data Collection

Data were extracted from electronic patient files. Data included demographic patient characteristics, cleft lateralization and extent, comorbidities, psychosocial diagnoses, the need for special education in the past or at present time, surgical procedures, postoperative complications, pre- and postsurgical detailed speech assessments, and information on the presence of hearing impairment in the past or at the present time. The retrieved speech assessments were reviewed by the speech pathologist who had performed the speech assessments. Presence of hearing impairment or hearing loss was determined by an otolaryngologist. If it was expected that the degree of hearing impairment could influence speech development and articulation, the patient was excluded from participation in the study.

Data collection and protection took place according to the privacy regulations of the tertiary care center. Since subjects are not being subjected to any handling, nor are there rules of human behavior being imposed, Institutional Review Board Approval was waived by the ethical committee of the hospital (MEC-2017-400).

### Speech Assessments

Speech was assessed at approximately 4 weeks before and 15 weeks after surgery. Speech evaluations were standardized and proceeded as follows by a single qualified speech-language pathologist who is closely involved in the cleft patients' care. All children had the same language background (Dutch) as the speech pathologist. Video recordings were collected using the same equipment and technique, with the examiner and camera in opposite direction of the individual.

The perceptual speech analysis was conducted before and after surgery according to the speech assessment protocol developed by Meijer and was based on reading standardized sentences and words. 16 These speech sounds were scored in several categories such as cleft-related articulation disorders, phonetic disorders (errors in positioning the tip of the tongue during production of alveolar fricatives), and phonological disorders not related to the cleft (the latter was not analyzed in this study). Only the cleft-related articulation disorder was further analyzed in this study and was scored as present or absent based on a general impression of the articulation ( Table 2 ).

**Table 2:** Speech assessment protocol used during the study period

Articulation disorders	Nasality	Hypernasality degree	Facial grimacing	Speech intelligibility
**1: Present** **2: Absent**	0: Normal 1: Hypernasal 2: Hyponasal 3: Mixed-type	1: Mild 2: Moderate 3: Severe	0: No grimacing 1: Nasal grimace 2: Nasal and midfacial grimace 3: Nasal, midfacial, and frontal grimacing	1: Intelligible speech 2: Speech differs, without commenting of others 3: Speech differs, with commenting of others 4: Hardly intelligible speech 5: No intelligible speech

To assess resonance (hypernasal, hyponasal, mixed-type), a subjective evaluation based on standardized words, vowels, sentences, and spontaneous speech was performed. For an objective assessment of nasal emission, the mirror test and nasometry (Nasometer II Model 6450 KayPENTAX) were used. In case of contradicting findings, the subjective nasality measurements would be conclusive since it is expected that this would also be the perception of the listener during everyday life. The resonance was scored based on the severity scale shown in Table 2 .

Speech intelligibility was rated using a five-point scale ranging from normal/intelligible speech to completely unintelligible speech ( Table 2 ). The score was based on spontaneous connected speech, lasting 2 to 5 minutes.

### Statistical Analysis

Statistical analyses are performed using the IBM Statistical Package for the Social Sciences (Version 21.0, SPSS Inc, Chicago, IL). For intergroup assessments over time (pre- and postoperative), Wilcoxon signed-rank test for ordinal variables and McNemar test for binary variables were used. To determine if variable outcomes were dependent on the cleft type (unilateral or bilateral), chi-square and Fisher's exact tests were used. A Spearman's test was used to determine correlations. Probabilities less than 0.05 were accepted as significant.

## Results

### Participants

Initially, a total of 51 patients with CLAP were eligible for this study, 3 patients were excluded due to lack of complete data. Eventually 48 patients, 33 with unilateral CLAP and 15 with bilateral CLAP, were included.

Of the 15 bilateral cases, 10 were bilaterally complete clefts, 3 were unilaterally complete, 1 was bilaterally incomplete, and in 1 patient the extent of cleft was unknown. The mean follow-up time was 10.1 (range 7–13) weeks, representing the time between the pre- and postoperative speech assessment.

### Patient Characteristics

Most patients were male and had a complete cleft ( Table 3 ). The cleft was determined as complete when it was anteriorly extended into the nasal floor. Nine patients (27%) were adopted and eight of these patients underwent primary closure of the lip and correction of the nose in the country of adoption. Five patients (10%) suffered from bilateral hearing loss in the past or at present time. These cases were reevaluated by an ENT specialist who concluded that speech disorders in these children were all associated with the cleft and were not related to their hearing impairment.

**Table 3:** Patient characteristics

*Variables*	*CLAP patients (n = 48)* *N (%)*
**Cleft side**	
Unilateral	33
Left	21 (64)
Right	12 (36)
Bilateral	15
**Sex**	
Male	28 (58)
Female	20 (42)
**Cleft extent**	
Complete	36 (75)
Incomplete	8 (17)
Right C, left IC	2 (4)
Left C, right IC	1 (2)
Unknown a	1 (2)
**Adopted**	
China	7 (15)
Philippines	1 (2)
Brazil	1 (2)

Abbreviations: C, complete; CLAP, cleft lip, alveolus, and palate; IC, incomplete.

Values are presented as number (%).

a Primary surgery in a different country with no documentation on the cleft extent.

### Surgical Treatment

DHPC and ABG were performed by two qualified oral and maxillofacial surgeons with extensive experience in cleft surgery. During the study period, the von Langenbeck palatoplasty was the preferred technique for closing the hard palate. This was also the case in bilateral cases. In all patients, autologous bone from iliac crest was harvested for alveolar repair.

The procedure was performed at the mean age of 10.5 (range 7–13) years. Two patients with unilateral CLAP suffered from postoperative complications in the alveolar area (fistula, bone sequestration) which needed surgical revision. One patient with bilateral CLAP developed a fistula in the anterior palate following synchronous closure. The palatal defect was closed 3 years later performing another von Langenbeck procedure. Velopharyngoplasty was performed in 15 patients before simultaneous DHPC and ABG at a mean age of 6.1 (range 2–9) years. This procedure was performed by plastic surgeons specialized in cleft surgery, according to the Orticochea technique (4 patients) or by using a cranial-based pharyngeal flap (11 patients). Two patients suffered from minor complications (small fistula, mucosal dehiscence) without a need for revision surgery, one had undergone velopharyngoplasty according to Orticochea technique, and another one had received a cranial-based pharyngeal flap. Two other patients were reoperated due to persistent VPI (one during and one after simultaneous DHPC and ABG).

Velopharyngoplasty (intravelar veloplasty) was performed in three patients after DHPC and ABG. Two of these patients were scored as severely hypernasal and one as moderately hypernasal both before and after DHPC and ABG. After conducting nasoendoscopy, VPI was found to be the cause of (severe) hypernasality which persisted after intravelar veloplasty in two of these patients. One patient underwent additional surgery for correction of VPI using cranial-based pharyngeal flap.

### Speech Outcomes

#### Resonance

Abnormal resonance (hypernasal, hyponasal, or a mixed-type) was observed in 45 of 48 patients (94%) preoperatively. An improvement was seen in a total of 19 patients (42%; *p* < 0.001) after surgery ( Table 4 ). Resonance was considered improved when preoperative hypernasal speech changed to normal, hyponasal, or mixed-type. This was due to the fact that speech is perceived as more intelligible when a hyponasal or mixed resonance is present compared with a hypernasal speech.

**Table 4:** Presence of articulation disorders and hypernasal speech preoperative versus postoperative

*Variable*	Present preoperative	Present postoperative	Improvement	*p* -Value a
*Cleft-related articulation disorder*	45 (94)	41 (85)	4 (8)	0.375
*Hypernasal speech*	43 (90)	26 (54)	19 (40) b	**<0.001**

Values are presented as number (%).

a McNemar test.

b Resonance was considered improved when hypernasal speech changed to hyponasal, mixed-type, or normal.

Hypernasal speech was found in 43 patients (90%) before and in 26 patients (54%) after surgical repair of the hard palate and alveolus ( Table 4 ). In 15 (58%) of these patients, hypernasal speech was scored as mild. Normal resonance was observed in 17 (35%) patients during postoperative evaluation, two of them presented with normal resonance also prior to surgery ( Table 5 ). The improvement in overall resonance did not differ between patients with unilateral and bilateral CLAP ( *p* = 0.171; Table 6 ).

**Table 5:** Postoperative changes in resonance in all 48 cleft lip, alveolus, and palate patients

*Change in resonance*	*N* umber *of patients*
*Hypernasal to normal*	15
*Remained hypernasal*	25
*Hypernasal to hyponasal*	3
*Hypernasal to mix* ed- *type*	1
*Mixed-type to hypernasal*	1
*Remained normal*	2

**Table 6:** Differences in improvement of speech disorders between unilateral and bilateral cleft lip, alveolus, and palate patients

Variable	*Unilateral CLAP (n = 33)*	*Bilateral CLAP (n = 15)*	* p-Value a*
*Articulation disorders*	1 (3)	3 (20)	0.227
*Hypernasality*	14 (42)	5 (33)	0.328
*Hypernasality degree*	21 (64)	8 (53)	0.499
*Facial grimacing*	8 (24)	5 (33)	0.509
*Speech intelligibility*	20 (61)	12 (80)	0.186

Abbreviation: CLAP, cleft lip, alveolus, and palate.

Values are presented as number (%).

a Chi-square test or Fisher's exact test.

The severity of hypernasal speech decreased in 29 patients (67%) at postoperative evaluation ( *p* < 0.001), remained unaffected in 16 patients (37%), and increased in 3 patients (7%). There was no difference in outcome regarding hypernasal speech or the severity grade between patients with unilateral and bilateral CLAP ( *p* = 0.498 and *p* = 0.499; Table 6 ).

#### Cleft-related Articulation Disorders

Cleft-related articulation disorders were present in 45 patients (94%). In four of these patients, articulation was restored during the follow-up period ( *p* = 0.375; Table 4 ). There was no difference in improvement of articulatory errors between patients with unilateral or bilateral CLAP ( *p* = 0.227; Table 6 ).

#### Facial Grimacing

The severity of facial grimacing decreased in 13 patients (27%) during the postoperative follow-up ( *p* = 0.015). In 33 patients (69%) no change was observed, in 2 patients (6%) the grimacing seemed to have increased from score 1 to 2. One of them had persistent hypernasality due to VPI and one developed mild hypernasal speech postoperatively. No difference was noted between patients with unilateral and bilateral CLAP ( *p* = 0.509; Table 6 ).

#### Speech Intelligibility

Intelligibility of speech improved in 32 patients (67%; *p* < 0.001). Intelligibility remained unchanged in 11 patients (23%) and speech was assessed as less intelligible in 4 patients (8%). There was no difference in the improvement of speech intelligibility between patients with unilateral and bilateral CLAP ( *p* = 0.186; Table 6 ). A positive correlation between the hypernasality degree and the intelligibility score was observed both pre- and postoperative (ρ = 0.419, *p* = 0.003 and ρ = 0.559, *p* < 0.001).

## Discussion

In this study, we aimed to determine the changes in resonance, articulatory patterns, and speech intelligibility in individuals with an isolated CLAP following a DHPC combined with secondary ABG between 9 and 12 years of age. Therefore, we evaluated 48 patients with unilateral and bilateral CLAP. We found a significant reduction in the number of patients with hypernasal speech following surgery. Additionally, in patients who had remaining hypernasal speech postoperatively, a significant decrease in severity of hypernasality was seen, which was mild in the majority of cases. No significant reduction in articulatory errors and grimacing (compensatory mechanisms) was seen. The speech intelligibility however was significantly improved during postoperative follow-up. No difference was found between the unilateral and bilateral group. Since the size of the bilateral group is relatively small, this group was not separately analyzed.

Within the past surgical cleft treatment protocol in our institution, according to which the studied cohort was treated, the focus of the treatment was minimizing the need for orthognatic surgery during adolescence by limiting the detrimental effects of early surgical procedures on the midfacial growth, while providing multidisciplinary care to achieve the best possible speech development. The philosophy on DHPC was based on earlier literature revealing that the most midfacial growth takes place before the age of 5 years and that irreversible injury after early surgery of the palate results in reduction of midfacial growth capacity. 17 18 19 A study of 251 patients who were subjected to the delayed two-stage palatoplasty in our institution reports a frequency of 11.2% of Le Fort I osteotomies which is relatively low compared with a frequency up to 70% reported by previous studies. 20 21 22 23 24 In the latter study, a higher number of previous surgical interventions was found in patients with an indication for orthognathic surgery compared with those without. 20

### Hypernasality

Hypernasality decreased from 90 to 54% after HPC and alveolar repair. The severity of hypernasal speech was reduced in 67% of patients so that in 31% of all patients only mild hypernasality was seen after surgery. Three patients were diagnosed with VPI prior to the surgery and were diagnosed with severe and moderate hypernasal speech which persisted postoperatively. All three of them were surgically treated for VPI multiple times after DHPC and ABG. Here, VPI was the main cause of hypernasality which decreased to mild and moderate scores following speech-enhancing procedures.

In 65% of patients postoperative compensatory facial grimacing persisted as mild, presenting as nasal valve flaring or contraction of the nasal bridge. Two patients presented with a higher score of facial grimace. In one of them, this could be explained by the severe VPI for which he was surgically treated multiple times after the delayed closure of the hard palate. One patient who did not present with preoperative grimace developed mild nasal contractions. The severity of hypernasal speech also increased which could be the reason for the occurrence of mild grimace.

In the study of Lohmander et al, speech outcomes of 55 Swedish patients treated according to a two-stage palatal closure with DHPC at a mean age of 8 years were evaluated at 5, 7, 10, 16, and 19 years. 25 A decrease of hypernasality was reported from 46% preoperatively at the age of 7 to 34% 2 years after DHPC. At 16 years, only 8% presented with hypernasal speech. These results are more favorable than our data present, which could be explained by a longer follow-up time or the functionally closed residual cleft in the hard palate in 29% of patients before 5 years.

Kappen et al evaluated speech in after HPC at 33 months of age. At 20 years of age still 38.6% of patients presented with hypernasal speech, compared with 58% hypernasality rate after DHPC in our study cohort within a 15-month follow-up period. 26 Yet another study reports a 40% remaining hypernasal speech at 10 years of age following a one-stage palatal closure at only 8 months. 27

These results suggest that DHPC does not necessarily result in unfavorable speech outcomes in terms of resonance. Since grimacing is a compensatory mechanism to reduce nasal air loss, we could expect a decrease of this habit in time when resonance improves. Reversing this habit would require awareness and training.

### Articulation Errors

In 85% of patients, articulation errors were noted after HPC. These errors were classified as only “present” or “absent,” even mild errors that occurred once during the assessment were scored as being abnormal. This can overestimate the articulatory disturbances that are clinically significant and notable during regular speech. No difference was observed between the unilateral and bilateral group.

Adaption of compensatory errors occurs when refined articulation placement, such as tongue tip placement is altered. 4 This could be due to the remaining cleft in the hard palate but could also occur as a result of dental malocclusion, palatal morphology, and anatomical irregularity of the palate and the alveolar ridge, more so in bilateral and larger defects, persisting even after surgery. 28 This is particularly true for alveolar and interdental articulation. 28 In the current study, the amount and type of articulation errors were not specified, and no records of dental occlusion were obtained; however, dental malocclusion is known to be a consequence of CLAP. 29 Haque et al 29 and Kappen et al 26 both found a 44 to 45% dental malocclusion in their cohort of unilateral CLAP. The demographic characteristics of our cohort are comparable to that in the latter Dutch study, therefore dental malocclusion could also partially account for the high prevalence of articulation disorders in our study.

Hortis-Dzierzbicka et al 27 assessed speech outcomes in 10-year-old children who were treated with one-stage closure at 8 months of age and found that compensatory articulatory patterns were present in only 4% of patients compared with 85% found in the current study. In the report by Noordhoff et al and a follow-up paper 23 years later, 30 31 all patients who underwent HPC later than 4 years of age presented with increased articulation errors, particularly those individuals with wide remaining cleft of the hard palate.

These results support the hypothesis that compensatory articulation errors are present in most patients with cleft palate and do not resolve immediately after HPC. 30 Although reversing these habits at a later age seem challenging, Lohmanderet al 25 reported a final decrease of articulation errors from 23% at 7 years to 6% at 10 years, 2 years after HPC. At 16 years of age, 96% of patients did not present any articulation disorders. At 19 years of age, only one individual was diagnosed to have mild articulation errors during speech. Additionally, Van Lierde et al 14 proved that postoperative speech therapy is essential to suppress or diminish the adapted compensatory articulation patterns. After previous early one-stage palatal closure or DHPC at 8 years of age, all patients had received at least 6 months of speech training. None of the subjects of their study presented with compensatory articulation disorders at 18 years.

### Intelligibility

Resonance and the amount of articulation errors is reported to play a role in intelligibility of speech. 14 25 26 Speech intelligibility increased from 35 to 71% (grade 1 and 2) during postoperative follow-up in the current study.

In four patients postoperative intelligibility seemed to be decreased. One of these patients had persistent hypernasality due to VPI which worsened over time and could account for the deterioration of intelligibility. The results of this study support a correlation between the decrease in hypernasal speech and the improvement in intelligibility, in accordance with the previous literature. 14 26

Lohmander et al reported an improvement of intelligible speech from 80% preoperatively at 7 years to 98 to 100% intelligible speech at 10 years of age, 25 compared with the 84% reported intelligible speech following much earlier HPC at 36 months reported by Kappen et al. 26

The literature indicates that timing of HPC is not the only factor contributing to better speech outcomes. Prior surgical procedures, their timing, and maybe more importantly sequential treatments such as adequate speech therapy combined with a good patient compliance can also play a key role in improvement of speech and reduction of compensatory mechanisms. 4 29 32 33

### Velopharyngeal Insufficiency

All patients in the current cohort underwent a soft palate closure between 9 and 11 months of age according to the Widmaier–Perko technique. This technique (Perko, 1979 34 ) results in a full-thickness linear midline scar in the soft palate due to transient ischemia of the periosteum followed by secondary epithelialization (Mommaerts, 2003 35 ). This fibrotic tissue could potentially be the cause of reduced mobility of the soft palate. Thirty-eight percent (18/48) of patients in this cohort were diagnosed with VPI and opted for a surgical repair. The primary closure of soft palate could partially account for persisting VPI weather or not confounded by a DHPC.

### Strengths and Limitations

This study gives insight into changes in resonance and compensatory mechanisms in speech after a two-stage palatoraphy including delayed closure of the hard palate between the age of 8 and 13 years. This is one of the few studies reporting these specific variables after HPC at the time of alveolar repair with the inclusion of bilateral cases. There are obvious limitations; mainly the retrospective design of the study and lack of data such as frequency and intensity of speech therapy. A longer postoperative follow-up time would have given more insight into the further development of articulatory changes following surgery. Furthermore, we included patients with bilateral cleft known for its higher anatomical variability demanding different surgical approaches. This might have impacted our results.

### Future Perspectives

The current cleft treatment protocol at our institution follows a more individualized approach to the timing of HPC. When the size of the defect of the hard palate technically allows it, closure will be performed at an earlier age. We have implemented a patient-reported set of outcome measurements, which includes patients' perspective of speech and psychosocial health throughout the treatment trajectory until their discharge from follow-up at the age of 22. 36 37 These data should give us more insight on the long-term patient-reported speech outcomes after DHPC.

### Conclusion

Based on the current speech outcome study in 48 patients with unilateral and bilateral CLAP, a significant improvement was seen in resonance, degree of hypernasality and intelligibility following a two-stage DHPC at the mean age of 10.5. However, habitual compensatory pattern remained during the follow-up period. Our data combined with previous literature suggest that treating ingrained compensatory errors does not only rely on anatomical repair and its timing but that postoperative speech therapy is crucial to reach optimal speech outcomes.
